# Can cost–effectiveness principles be ignored in urgent times?

**DOI:** 10.2217/cer-2021-0211

**Published:** 2021-10-20

**Authors:** Zoltán Kaló, Bertalan Németh, Antal Zemplényi

**Affiliations:** ^1^Center for Health Technology Assessment, Semmelweis University, Üllői Street 25, Budapest, 1091, Hungary; ^2^Syreon Research Institute, Mexikói Street 65/A, Budapest, 1142, Hungary; ^3^Division of Pharmacoeconomics, Faculty of Pharmacy, University of Pécs, Rákóczi Street 2, Pécs, 7623, Hungary

**Keywords:** coverage with evidence development, COVID-19, evidence-based decision-making, health economics, real-world data

## Managing uncertainty in health technology assessment

As part of the health policy tools, health technology assessment (HTA) is most commonly used to judge the clinical and economic value of new technologies or existing technologies in new indications in order to support pricing and reimbursement decisions. At different levels of sophistication HTA has already become a standard prerequisite of policy decisions in many countries [1]. During the last decades public reports by prestigious HTA bodies are more and more regularly referenced in many different countries and publications; hence, it can be concluded that mandatory HTA requirement across jurisdictions has improved the evidence base of new health technologies globally.

In the technology assessment process, there are standard methods (e.g., sensitivity analysis of economic models) to deal with the uncertainty in the evidence for clinical and economic outcomes [2]. Still, the evidence base of certain technologies can be highly limited in the early periods after market authorization. In such cases, decision makers are confronted with the trade-off between delaying reimbursement of a potentially valuable technology due to premature scientific evidence or adopting a new technology that over time may be less safe, effective or cost-effective than expected.

In areas of high unmet need, it is desirable to provide timely access to promising treatments, even if the scientific evidence is not yet mature, as seen in the case of orphan medicines [3]. While it is not justifiable to delay policy decisions in priority areas, it is also not reasonable to ignore the implications of policy decisions. Methods have been introduced to reduce this uncertainty and allowing temporary, conditional reimbursement while additional data are collected and assessed, for example, coverage with evidence development (CED) [4].

## Pandemic periods – a special case of uncertainty

Pandemic situations (for example: SARS, Ebola and COVID-19) are special, as waiting for better evidence base to judge the true value of potentially beneficial technologies put risk on the spread of infections, which can have detrimental implications not only on the health of infected patients. Due to the exhaustion of healthcare capacities, all patients with other serious acute or chronic diseases may suffer the consequences [5]. Furthermore, pandemics have huge societal implications, the whole economy can be compromised [6,7].

The COVID-19 pandemic highlighted the need to make policy decisions on the adoption of new technologies or repurposing of existing technologies without relying on robust evidence, despite obviously huge health, economic and societal implications. This occurred because enormous pressure was coming from the general public and the media as well, as seen in the cases of the introduction of newly developed COVID vaccines to control the spread of infection or use of medical technologies, (such as different ventilation techniques) or repurposed medicines (such as hydroxychloroquine, remdesivir, favipiravir or ivermectin) to treat infected patients.

Unfortunately, in the overwhelming majority of cases worldwide neither the rapid HTA process nor CED schemes have been applied or developed to facilitate the value judgement of COVID vaccines or therapies. Healthcare payers and HTA bodies were not prepared to take rapid decisions to avert the emergency caused, and sometimes even when there was a chance to rely on their judgements, high-level decision makers disregarded expert advice from HTA professionals [8].

This does not change the fact, that decisions on the procurement and/or widespread use of technologies (vaccines, repurposed medicines, ventilators, etc.) have opportunity costs, even if these were not taken into account due to the enormous time pressure.

## Recommendations

In order to overcome these complex issues, we compiled a set of recommendations. We believe that instead of upfront value judgement of individual health technologies, at first the cost–effectiveness of the policy approach should be considered. We believe that even under extreme circumstances, decision makers should adhere to the key principles of what is considered to be the good practice of evidence-based decision making as much as they can [9]. Therefore, in pandemic situations the health economics approach, whereby the opportunity costs are taken into account, should be extended beyond the evaluation of individual technologies to the evaluation of decision strategies by comparing three main alternative approaches, including: coverage without evidence development; wait for better evidence and delay intervention; and coverage with evidence development. The decision on the use of individual technologies should only be made only once this decision has been taken.

It should be borne in mind that similar decisions will likely have to be made in the near future, as COVID-19 may not be the last global or regional pandemic. Furthermore, when making rapid policy decisions, it should be kept in mind that the decision will be reviewed later and scrutinized not only by the healthcare professionals, but in eyes of the general public as well. Therefore, we not only believe that future decisions should build on the experiences of previous decisions, but that collection of real-world data should be part of the policy package when making rapid decisions. Hence, procurement decisions or development of local therapeutic guidelines must be supplemented by a design of data collection methodology, which can be used to generate real-world evidence in the future.

Emerging evidence about effectiveness and safety of new or repurposed technologies should be considered not only a public good, but global public good, because health and economic problems of other countries in pandemic situations cannot be stopped at the borders of individual countries. We believe it is unacceptable to hide emerging data about the comparative effectiveness and safety of vaccines for specific subpopulations or against specific viral variants in the times of global health crisis [10].

As we are writing this paper, the pandemic has been ravaging through the world for more than 18 months. If setting up a robust decision-making framework is constantly delayed on the grounds that it cannot answer the question of that particular week, it will never happen. If experts had been able to start developing a framework at the beginning of the pandemic, it would likely be operational by now. Although we recognize the practical challenges of this task, our recommendations are a call to action for health policy makers to develop frameworks for data collection, make data publicly available and review previous decisions based on real-world evidence.

## Conclusion

Making procurement and reimbursement decisions in urgent times without insisting on collecting data and developing new evidence creates a lost opportunity in the future because the valuable information to make necessary adjustments is not available and the price of an inefficient decision must be paid over a longer period than necessary. When the spread of the infection or maintaining the viability of the economy is at stake, it is not an appropriate decision to wait for further evidence. As a reasonable and scientifically sound compromise, CED schemes offer an opportunity for reducing this uncertainty in these situations by allowing temporary reimbursement while additional data are collected and assessed. As such, CED schemes facilitate final reimbursement decisions at a later stage without delaying patient access to therapies or vaccines.

Some unique features should be considered in designing CED schemes in pandemic periods. At first, cost of real-world data collection and its burden on healthcare professionals should be minimized. On the other hand, real world data on both surrogate and hard end points are generated fairly quickly in pandemia, and governments usually ease restrictions to aggregate data without prior consent of patients. Therefore, CEDs can (and should) be built on existing data with special focus on linking patient records in different databases, including epidemiological data collected by disease control centers, centralized vaccination data, electronic medical records at healthcare providers, resource utilization and cost data in payer’s database and other potentially relevant nonmedical data (e.g., population movement monitoring data based on mobile phone usage) [11]. When additional information is needed to supplement routinely collected data, evidence generation should be handled within the existing infrastructure with minimal burden on exhausted healthcare providers.

We believe that the role of health economists is to respond to the new situation created by the pandemic and come up with proposals on how decision frameworks can be improved. If health economists will not speak up to be the voices of reason, individuals less qualified in the subject or politicians (with vested interest to support their own decisions or criticize decisions made by others) will take their place, spreading their own suboptimal views on the subject.
